# Visual Performance and Quality of Life after Femtosecond Laser-Assisted Cataract Surgery with Trifocal IOLs Implantation

**DOI:** 10.3390/jcm10143038

**Published:** 2021-07-08

**Authors:** Carla Enrica Gallenga, Rossella D’Aloisio, Erminia D’Ugo, Luca Vecchiarino, Luca Agnifili, Maria Beatrice Simonelli, Marta Di Nicola, Lisa Toto, Paolo Perri

**Affiliations:** 1 Department of Medical Sciences, University of Ferrara, 44121 Ferrara, Italy; gllcln@unife.it; 2 Department of Medicine and Aging Science, Ophthalmology Clinic, University “G. d’Annunzio” of Chieti-Pescara, 66100 Chieti, Italy; erminia.dugo@gmail.com (E.D.); l.vecchiarino@unich.it (L.V.); l.agnifili@unich.it (L.A.); mbeatrice.simonelli@gmail.com (M.B.S.); l.toto@unich.it (L.T.); 3 Laboratory of Biostatistics, Department of Experimental and Clinical Sciences, University “G. d’Annunzio” of Chieti-Pescara, 66100 Chieti, Italy; mdinicola@unich.it; 4 Section of Ophthalmology, Department of Neuroscience and Rehabilitation, University of Ferrara, 44121 Ferrara, Italy; paolo.perri@unife.it

**Keywords:** trifocal IOLs, femtosecond laser-assisted cataract surgery, quality of life

## Abstract

Purpose: To assess visual performance and quality of life after implantation of diffractive trifocal IOLs with enhanced depth of focus (Acriva Reviol Tri-ED) compared to monofocal IOLs. Setting: Ophthalmology Clinic, Department of Medicine and Science of Ageing, University “G. d’Annunzio” Chieti-Pescara, Italy. Design: Prospective clinical study. Methods: This study comprised 36 eyes of 18 patients with senile cataract candidates for phacoemulsification and implantation of the Acriva Reviol Tri-ED (Group 1–18 eyes) and the AcrySof IQ Monofocal IOL SN60WF (Group 2–18 eyes). The main outcome measures, over a 6-month follow-up period, were uncorrected and corrected visual acuity at different distances (40, 60 cm and 4 m), defocus curve, contrast sensitivity and wavefront error. Patient satisfaction was evaluated by means of the NEI-RQL-42 questionnaire. Results: At 180 days postoperatively, the difference of the UCDVA and CDVA between the groups was not statistically significant (*p* = 0.888 and *p* = 0.843, respectively). The difference between the groups was statistically significant for UCIVA (*p* = 0.019) and UCNVA (*p* = 0.036). The mean values of contrast sensitivity under photopic and mesopic conditions were not significantly different between the groups. The RMS of spherical aberration was significantly lower in Group 1 compared to Group 2. The NEI-RQL-42 questionnaire showed statistically significant differences between the groups for the dependence on correction (*p* < 0.001). Conclusions: The diffractive trifocal IOL with enhanced depth of focus Acriva Reviol Tri-ED was effective in improving functional capacity for intermediate and near vision compared to monofocal IOLs and provided a good quality of vision due to a significant reduction in spherical aberration.

## 1. Introduction

The aim of intraocular lenses (IOLs) implantation is to provide a high quality of visual performance and to increase spectacle independence. Monofocal IOLs designs supply either near or distance vision, so multifocal IOLs (MIOLs) with refractive or diffractive optical designs, or the combination of both, have been introduced to overcome this limitation, allowing the treatment of presbyopia and decreasing spectacle dependence. Nowadays, many usual activities, such as the use of tablets or computers, need good intermediate vision. While traditional MIOLs provide good near and distance vision but cause difficulty in intermediate vision, trifocal IOLs supply an improvement in intermediate vision without impairing distance and near visual acuity [1].

The implant of MIOLs may be related to unpleasant visual symptoms such as halos, glare and loss of contrast sensitivity.

Recently, technological advancements have been introduced to solve these issues: asphericity of the optics, apodization, refractive rotational asymmetry, lower addition for near vision, trifocal MIOLs, and enhanced depth of focus technology. These technological innovations partially or totally solve these concerns and improve quantity and quality of vision, thus increasing patient satisfaction and quality of life [2,3].

Recently, a new intraocular lens (Acriva Reviol Tri-ED, VSY Biotechnology, Istanbul, Turkey), which combines trifocal diffractive technology with an enhanced depth of focus property, has been developed to improve the visual performance of patients. In our study, we used it in a group of patients to evaluate visual performance (distance, intermediate and near visual acuity), contrast sensitivity, wavefront error and quality of life compared to monofocal IOLs (Acrysof SN60WF, Alcon Laboratories, Inc., Fort Worth, TX, USA).

## 2. Materials and Methods

This prospective nonrandomized clinical study adhered to the tenets of the Declaration of Helsinki. The protocol was approved by the Institutional Review Board of University “G. d’Annunzio” of Chieti-Pescara (ethical approval code: TriED_5/2020). The setting of the trial was the Ophthalmology Clinic, Department of Medicine and Science of Ageing, University “G. d’Annunzio” Chieti-Pescara, Italy.

This study comprised 36 eyes of 18 patients with senile cataracts, candidates for phacoemulsification and implantation of an IOL, who were divided into 2 groups: Group 1 (9 patients, 18 eyes) was implanted bilaterally with a diffractive trifocal IOL with enhanced depth of focus (Acriva Reviol Tri-ED); Group 2 (9 patients, 18 eyes) was implanted bilaterally with a monofocal IOL (Alcon Acrysof SN60WF).

The inclusion criteria were bilateral age-related cataracts, preoperative corneal astigmatism less than 1.0 diopter (D) and age of more than 45 years.

The exclusion criteria were history of previous ocular surgery, presence of other eye diseases (ocular trauma, glaucoma, diabetic retinopathy, PEX, chronic uveitis and corneal opacity), fundus oculi alterations and intraoperative complications.

Before cataract surgery, patients had a complete ophthalmologic examination including manifest refraction; distance, intermediate and near visual acuity; topography and wavefront analysis using OPD- Scan II (NIDEK Co. Ltd., Gamagori, Japan); slit-lamp biomicroscopy; tonometry; and fundus ophthalmoscopy after pupil dilation.

IOLMaster (Carl Zeiss Meditec, Jena, Germany) was used to measure axial length and to calculate IOL spherical power using the SRK/T formula with targeted refraction for emmetropia.

The Acriva Reviol Tri-ED combines trifocal diffractive technology with enhanced depth of focus property. Intraocular lens features are reported in the manufacturer’s documents [4].

## 3. Surgical Technique

Femtosecond laser-assisted cataract surgery (FLACS) was performed for all patients by the same surgeon using topical anesthesia. A three-plane primary clear corneal incision, a secondary monoplanar corneal incision, capsulotomy and lens fragmentation were made using the Catalys precision laser system (Abbott Medical Optics, Santa Ana, CA, USA).

Standard phacoemulsification with a 2.2 mm tip and sleeve was used to complete the surgery using the Alcon Constellation System (Alcon Laboratories, Inc., Fort Worth, TX, USA).

All intraocular lenses were implanted in the capsular bag. Ofloxacin 0.3% and dexa- methasone 0.2% eye drops were administered four times daily for 3 weeks.

Patients were examined over a 6-month follow-up period.

The main outcome measures were spherical equivalent (SE) manifest refraction, uncorrected and corrected distance visual acuity (UCDVA-CDVA), uncorrected and corrected intermediate VA at 60 cm (UCIVA-CIVA), uncorrected and corrected near VA at 40 cm (UCNVA-CNVA), contrast sensitivity under photopic (85 cd/m^2^) and mesopic conditions (3 cd/m^2^), wavefront analysis and quality of life by means of the National Eye Institute Refractive Error Quality of Life instrument (NEI-RQL-42 questionnaire).

All evaluations were performed before surgery and at 1, 30, 90 and 180 days after surgery.

## 4. Visual Performance Assessment

Monocular uncorrected and corrected distance visual acuities were measured at 4 meters using ETDRS (Early Treatment Diabetic Retinopathy Study) charts.

Monocular uncorrected and distance-corrected near visual acuities were examined at 40 cm using a hand-held ETDRS near reading chart.

Monocular uncorrected and distance-corrected intermediate visual acuities were evaluated at 60 cm using the same chart used for near assessment.

The visual performance and the defocus curve assessments were performed as previously described [5].

## 5. Contrast Sensitivity Evaluation

Contrast sensitivity was evaluated monocularly at 1.05, 3, 6, 12 and 18 cycles per degree (cpd) under photopic (85 cd/m^2^) and mesopic (3 cd/m^2^) conditions by means of Vistech contrast sensitivity charts displayed by the Yang vision tester (SIFI Diagnostic SPA, Treviso, Italy).

## 6. Wavefront Aberration Analysis

The measurement of HOAs was performed by OPD-Scan II. The wavefront aberration analysis was assessed as previously described [6].

## 7. Quality of Life Assessment

To evaluate patient satisfaction and spectacle independence, all patients completed the Italian version of the National Eye Institute Refractive Error Quality of Life Instrument-42 (NEI RQL-42) questionnaire during the 3- and 6-month follow-up visits.

The NEI-RQL-42 is a questionnaire used to measure refractive error-related quality of life (QoL). The scored questionnaire consists of 42 items (questions) across 13 subscales.

Only 5 subscales were considered in our study: near vision, activity limitations, dependence on correction, appearance and satisfaction with correction.

## 8. Statistical Analysis

The quantitative variables were summarized as mean and standard deviation (SD). Qualitative variables were summarized as frequency and percentage. The Shapiro–Wilk test was performed to evaluate the departures from normality distribution for each variable.

Differences between groups in gender were compared using Fisher’s exact test. The Student *t* test for unpaired data was performed to evaluate differences in quantitative parameters between groups.

An analysis of variance (ANOVA) for repeated measures was performed to evaluate the effect of time (baseline vs different time point), group (Acriva vs. Monofocal) and their interaction on visual acuity parameters.

The false discovery rate correction (FDR) was used to control the family-wise type I error rate, and an FDR adjusted *p* value less than 0.05 was determined to be statistically significant. *p*-values below 0.05 were considered statistically significant. Statistical analysis was performed using IBM^®^ SPSS Statistics v 20.0 software (SPSS Inc., Chicago, IL, USA).

## 9. Results

The study comprised 36 eyes of 18 patients, divided into two groups.

Group 1 (9 patients, 18 eyes) was implanted bilaterally with a diffractive trifocal IOL with enhanced depth of focus (Acriva Reviol Tri-ED); Group 2 (9 patients, 18 eyes) was implanted bilaterally with a monofocal IOL (Alcon Acrysof SN60WF).

Table 1 shows the patient characteristics. Statistically significant differences between the two groups were not observed.

In Group 1 there were five men and four women; the mean age was 55.5 ± 5.6 years (range 49 to 63 years). In Group 2 there were five men and four women; the mean age was 54.4 ± 5.3 years (range 47 to 62 years). The between-group difference in age and gender was not statistically significant (Table 1).

### 9.1. Visual Parameters

Visual parameters are shown in Table 2.

**Figure 1:**
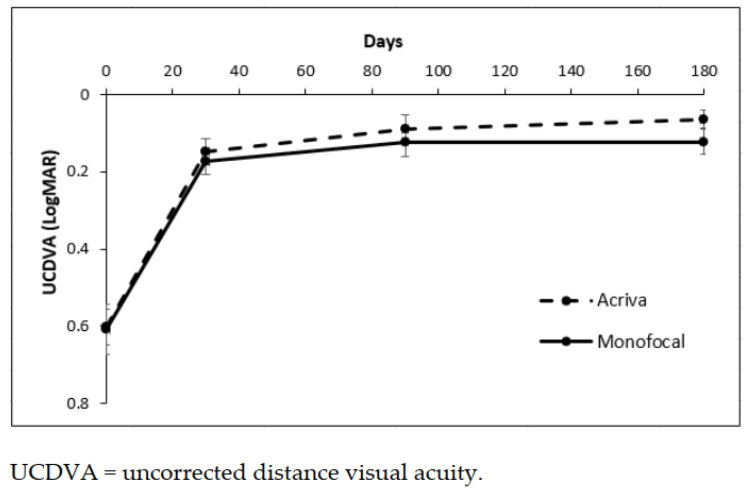
Mean and standard error of UCDVA in the two groups.

At 6 months, the difference between the UCDVA and CDVA between the groups was not statistically significant (*p* = 0.888 and *p* = 0.843, post-hoc analysis). The difference between preoperative and postoperative UCDVA was statistically significant in both groups (*p* < 0.001) (Figure 1).

Figure 1 shows the mean and standard error (SEM) of UCDVA in two groups, *p* < 0.001 time effect, *p* = 0.469 type of IOL effect, and *p* = 0.469 interaction term of the ANOVA test for repeated measures.

**Figure 2:**
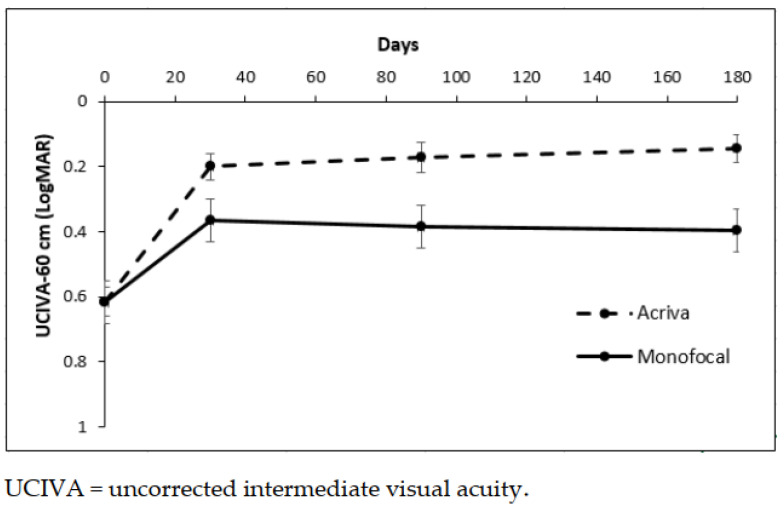
Mean and standard error of UCIVA at 60 cm in two groups.

At 6 months, the UCIVA was 0.12 ± 0.18 logMAR in Group 1 and 0.39 ± 0.14 logMAR in Group 2. The difference between the groups was statistically significant (*p* = 0.019). The difference over the time of UCIVA was statistically significant (*p* < 0.001) and different between groups (*p* = 0.0149 interaction term) (Figure 2).

Figure 2 shows the mean and standard error (SEM) of UCIVA at 60 cm in two groups, *p* < 0.001 time effect, *p* = 0.019 type of IOL effect, and *p* = 0.049 interaction term of the ANOVA test for repeated measures.

**Figure 3:**
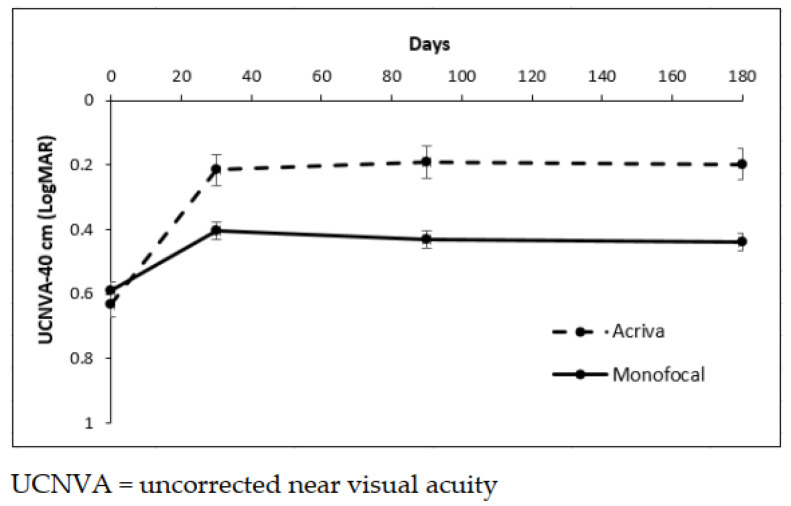
Mean and standard error of UCNVA at 40 cm in two groups.

At 6 months, the UCNVA was 0.17 ± 0.21 logMAR in Group 1 and 0.43 ± 0.11 logMAR in Group 2. The difference between the groups was statistically significant (*p* = 0.036). The difference over the time in UCNVA was statistically significant (*p* < 0.001), but there was a significant difference between groups (*p* = 0.036) (Figure 3).

Figure 3 shows the mean and standard error (SEM) of UCNVA at 40 cm in the two groups, *p* < 0.001 time effect, *p* = 0.036 type of IOL effect, and *p* = 0.057 interaction term of the ANOVA test for repeated measures.

The CNVA was 0.01 ± 0.03 logMAR in Group 1 and 0.00 ± 0.02 logMAR in Group 2, without statistically significant difference between the groups at 6 months (*p* = 0.211).

### 9.2. Refractive Parameters

The spherical equivalent (SE) at 6 months was −0.10 ± 0.14 D and −0.07 ± 0.12 D in Groups 1 and 2, respectively. The difference between the groups was not statistically significant (*p* = 0.460).

The postoperative astigmatism was −0.19 ± 0.27 D and −0.15 ± 0.24 D in Groups 1 and 2, respectively. The difference between the groups was not statistically significant (*p* = 0.615).

### 9.3. Contrast Sensitivity (CS)

**Figure 4:**
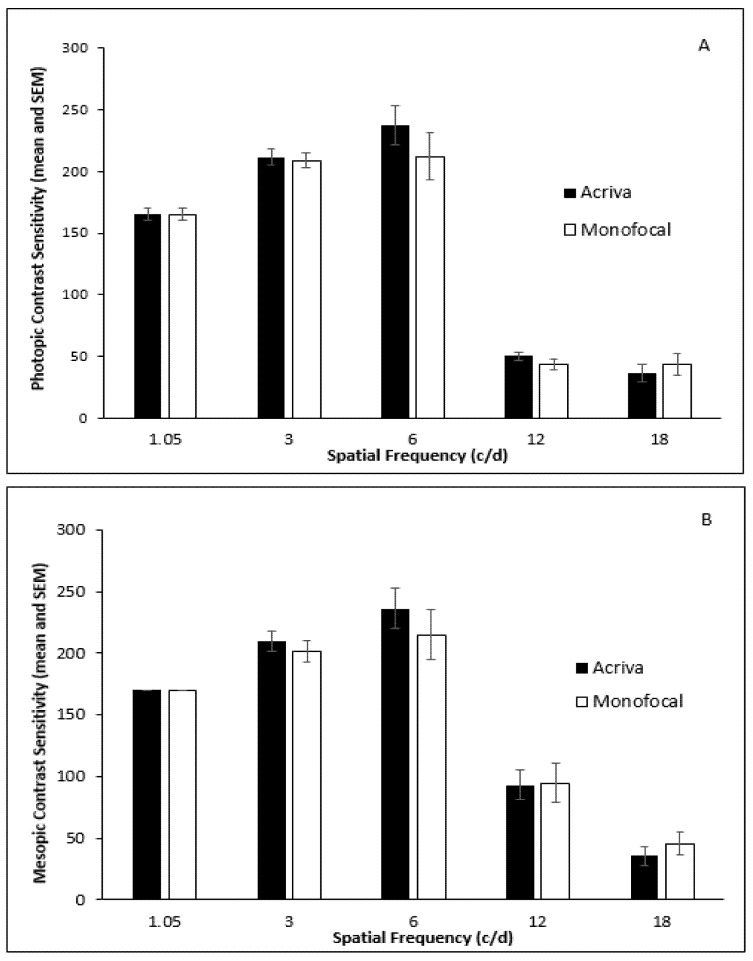
Contrast sensitivity under photopic (**A**) and mesopic (**B**) conditions in two groups.

Contrast sensitivity was evaluated under photopic (85 cd/m^2^) and mesopic (3 cd/m^2^) conditions by means of Vistech contrast sensitivity charts displayed by the Yang vision tester (SIFI) at various spatial frequencies (1.05, 3, 6, 12 and 18 cycles per degree-cpd). The mean values of contrast sensitivity at all spatial frequencies under photopic and mesopic conditions did not show a statistically significant difference between the groups (Figure 4).

### 9.4. Defocus Curve

**Figure 5:**
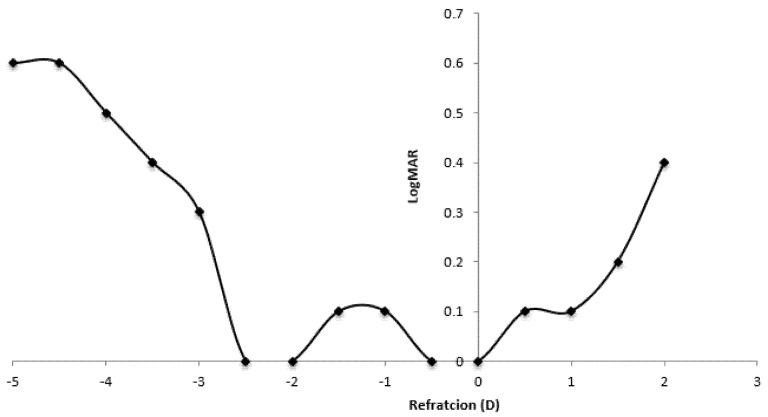
Binocular defocus curve in patients.

The mean visual acuities for different defocus values are demonstrated in Figure 5.

The depth of focus was 4.5 diopters in Group 1 and 1.5 diopters in Group 2.

In the present study, the defocus curve obtained by trifocal IOL showed a tendency of VA preserved at all distances.

## 10. Wavefront Aberration Analysis

The aberrometric parameters are shown in Table 3.

The RMS of HOA, coma, trefoil and spherical aberrations significantly decreased over time, and spherical aberration was significantly lower in the ACRIVA group compared to the monofocal group (Table 3).

## 11. Quality pf Life Assessment

Patient satisfaction was evaluated at 90 and 180 days by means of the NEI RQL-42 questionnaire.

Evaluation of the NEI RQL-42 questionnaire showed statistically significant differences between the groups for the dependence on correction (*p* < 0.001) at 6 months, with better results in Group 1.

**Figure 6:**
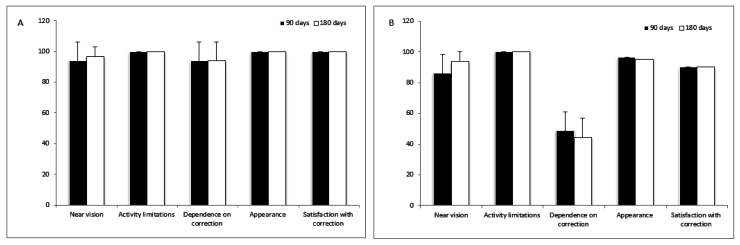
NEI-RQL-42 questionnaire in two groups at different time points.

Statistically significant differences were not found for near vision, activity limitations, appearance and satisfaction with correction (Figure 6).

Figure 6 shows the NEI-RQL-42 questionnaire in two groups at different time points (90 and 180 days). Panel A shows ACRIVA, and Panel B shows monofocal.

## 12. Discussion

The primary aim of multifocal IOL implantation is providing a high quality of vision and spectacle independence for vision at all distances.

With the use of traditional multifocal IOLs, successful levels of distance and near visual acuities have been obtained, but not of intermediate VA. Several authors demonstrated a near visual performance higher than J3 in between 92% and 99% of patients implanted with diffractive and refractive MIOLs, with better values in diffractive MIOLs [5,7,8,9].

Nevertheless, the multifocal IOL design can lead to an impairment of image clarity due to the generation of different overlapping images through several foci, thus causing a reduction in image sharpness, especially at low-contrast conditions, and visual symptoms such as halos, glare and blurred far vision [10,11].

Moreover, the balance between the negative spherical aberration of the crystalline and the positive spherical aberration of the normal cornea disappears after cataract surgery, causing an increase in the overall positive spherical aberration of the eye [12].

Recently introduced technical innovations in MIOLs technology, such as asphericity of the optics, apodization, refractive rotational asymmetry, lower addition for near vision, trifocal MIOLs and enhanced depth of focus technology, have improved the quantity and quality of vision of post-cataract-surgery patients, thus increasing patient satisfaction and quality of life [1,2].

The introduction of trifocal IOLs aims at overcoming the limitations of traditional bifocal IOLs. Their use has significantly improved intermediate VA, without impairing near and distance vision, thus enhancing patient satisfaction and quality of life [13,14].

In our study we assessed visual performance at all distances and wavefront error in patients bilaterally implanted with the diffractive aspheric trifocal IOL with enhanced depth of focus Acriva Reviol Tri-ED (Group 1) and compared to results obtained in patients bilaterally implanted with the monofocal aspheric Acrysof SN60WF IOL (Group 2).

At 6 months, both groups of patients showed excellent distance vision, with a mean UCDVA of 0.06 ± 0.09 logMAR in Group 1 and 0.12 ± 0.13 logMAR in Group 2, and a mean BCDVA of 0.02 ± 0.05 logMAR in Group 1 and −0.01 ± 0.04 logMAR in Group 2, with no statistically significant difference. At 6 months, UCIVA and UCNVA significantly increased in Group 1, while no statistically significant difference was found in Group 2. These results showed that the trifocal IOL used in this study is very effective in providing the best visual acuities at all distances. Torun Acar et al. [15] obtained similar results using the same trifocal IOL in 80 eyes of 40 patients, with UCDVA, UCIVA and UCNVA values at 6 months of −0.04 ± 0.08, 0.08 ± 0.11 and 0.15 ± 0.12 logMAR, respectively.

Studies on different commercial models of trifocal IOLs have reported good distance, intermediate and near VAs [16,17,18,19,20,21,22,23,24,25].

In their studies, Carballo-Alvarez et al. [16], Sheppard et al. [17], Vryghem and Heireman [18] and Cochener et al. [19] performed FineVision trifocal IOL implantation in patients with cataracts and reported good distance, near, and intermediate VAs with high patient satisfaction and a high spectacle independence. Kohnen et al. [20], Kretz et al. [21,22] and Mojzis et al. [23] evaluated visual performances after AT LISA trifocal IOL implantation and reported high levels of VAs for all distances. In their studies, Lawless et al. [24] and García-Pérez et al. [25] evaluated uncorrected visual acuity at far, intermediate and near distances after implantation of the Acrysof IQ Panoptix IOL and obtained results equivalent with other trifocal IOLs outcomes. We also found that the uncorrected distance, intermediate and near visual acuities 6 months after binocular implantation of the Acriva Reviol Tri-ED were high and similar to those described for other trifocal lenses. Uncorrected distance visual acuity was similar or slightly worse in our study (0.06 ± 0.09 logMAR) than reported for the AT LISA Tri by Kohnen et al. [20] (0.01 ± 0.11 logMAR), for the FineVision Trifocal IOL by Vryghem et al. [18] and Cochener et al. [19] (−0.04 ± 0.09 and 0.02 ± 0.09 logMAR, respectively), and for PanOptix IOL by Lawless et al. [24] (0.01 ± 0.10 logMAR). Intermediate visual acuity was similar for the Acriva Reviol tri-ED (0.12 ± 0.18 logMAR) to the values reported for the AT LISA Tri by Kretz et al. [22] (0.10 logMAR or better) and for the PanOptix by García-Pérez et al. [25] (0.12 logMAR), and slightly worse than reported for the FineVision by Vryghem et al. [18] and Cochener et al. [19] (−0.10 ± 0.15 and 0.05 ± 0.08 logMAR, respectively). Near visual acuity (0.17 ± 0.21 logMAR) was similar to that reported by Lawless et al. [24] (0.18 ± 0.10 logMAR), but worse than that reported by García-Pérez et al. [25] (0.02 ± 0.09 logMAR) for the PanOptix, and slightly worse than that reported for the AT LISA Tri by Kretz et al. [22] (0.10 logMAR or better) and for the FineVision by Vryghem et al. [18] and Cochener et al. [19] (0.02 ± 0.06 and 0.00 ± 0.04 logMAR, respectively).

In the present study, the best levels of contrast sensitivity were achieved at low (1.05, 3 cpd) and medium (6 cpd) spatial frequencies. Torun Acar et al. [15], Vryghem and Heireman [18], and Kretz et al. [22] also obtained high contrast sensitivity at 3 cpd. Mojzis et al. [13] and Sheppard et al. [17] achieved the best level of contrast sensitivity at 6 cpd.

A low wavefront error was found in Group 1, with the RMS of total, HOA, coma, trefoil and spherical aberrations significantly lower in Group 1 than in Group 2. Mojzis et al. [26] did not find statistically significant differences between ocular aberrations in patients implanted with diffractive bifocal IOL (AT LISA 801) and diffractive trifocal IOL (AT LISA tri 839 MP). Hida et al. [27] in their study found that values for total and spherical aberrations in the multifocal group (implanted with AcrySof SN60D3) were statistically lower than in the monofocal group (AcrySof SN60AT). Monaco et al. [28] compared two diffractive MIOLs, a trifocal IOL (PanOptix) and an extended-range-of-vision IOL (Symfony), with a monofocal IOL (SN60WF); they found that total HOAs and intraocular HOAs at a 3.0 mm pupil diameter were not statistically different between the three groups, while at a 5.0 mm pupil diameter the HOAs increased in the multifocal IOL groups, with higher values in the extended-range-of-vision group.

In any case, some studies demonstrated that the accuracy of wavefront aberration measurements was limited with diffractive multifocal IOLs [29,30], and in particular the Hartmann–Shack (H-S) aberrometer of the OPD-Scan II System used in our study was possibly unable to detect the highest-order aberrations induced by the diffractive components of the evaluated trifocal IOL. Moreover, H-S sensors are not designed to capture the scattering incurred by the discrete junctions between the diffractive zones, and this may lead to an overestimation of the optical quality of eyes implanted with diffractive MIOLs. However, we found lower amounts of aberration than those reported for eyes implanted with monofocal IOLs. This might be in relation with specific optical properties of the evaluated IOL, such as aspheric optic design and semi-apodization.

These objective results, which demonstrated a high quality of vision in patients implanted with Acriva Reviol Tri-ED, were confirmed by a subjective evaluation of visual performance by means of measurement of contrast sensitivity and visual acuity that provided indirect information about the total optical degradation of the eye.

In our study the defocus curve at 6 months showed an effective improvement of intermediate VA in addition to near and distance VAs. This result was consistent with the finding of Torun Acar et al. [15], who studied the same trifocal IOL. Various studies suggested that trifocal IOLs tended to perform better than bifocal IOLs, especially at the intermediate distance, although both groups showed a decline in VA at that distance [26,31,32,33,34].

In the present study, a high level of satisfaction was achieved in both groups for activity limitations, near vision, appearance and satisfaction with correction, with better results in Group 1 for the last three subscales. Group 1 achieved a higher level of satisfaction for the dependence on correction than Group 2, thus confirming the greater spectacle independence in patients implanted with trifocal IOLs. High satisfaction after implantation of trifocal IOLs was reported in other studies [31,32,33,34]. In their study, Jonker et al. [32] reported that spectacle independence was achieved more frequently with trifocal than with bifocal IOL implants.

The particular IOL design of Acriva Reviol Tri-ED explains the excellent outcomes achieved in our study. In fact, it presents technical features (such as trifocal structure associated with EDOF technology, high light transmittance, optimum light distribution, appropriate intermediate and near sight additions, semi-apodization and aspheric optic design) that enable it to overcome the problems of bifocal IOLs, thus improving visual performance and quality of life.

## 13. Conclusions

In conclusion, the diffractive trifocal IOL with enhanced depth of focus Acriva Reviol Tri-ED appears to be an appropriate option for patients motivated to obtain true spectacle independence. It is able to provide effective visual performance at all distances after cataract surgery, with a high level of quality of vision and patient quality of life.

## Figures and Tables

**Table 1:** Preoperative characteristics of the two groups.

	Group		*p*-Value ^a^
Parameter	ACRIVA	Monofocal	
Gender (M/F)	5/4	5/4	1000
Age (y)	55.5 ± 5.6	54.4 ± 5.3	0.205
Preoperative CDVA	0.4 ± 0.2	0.5 ± 0.2	0.937
K1 (D)	43.0 ± 0.4	42.9 ± 0.4	0.309
K2 (D)	43.5 ± 0.3	43.4 ± 0.4	0.479
Q value	−0.20 ± 0.08	−0.19 ± 0.07	0.669
Scotopic pupil size (mm)	5.2 ± 0.3	5.2 ± 0.9	0.613
Mesopic pupil size (mm)	4.4 ± 0.6	5.1 ± 0.4	0.201
Photopic pupil size (mm)	2.8 ± 0.3	3.4 ± 0.4	0.594

CDVA = corrected distance visual acuity; K = keratometry; D = diopters. ^a^ Differences in gender were compared using Fisher’s exact test. All other statistical comparisons were performed with two-tailed Student’s *t* test for unpaired data. Data are expressed as mean ± standard deviation.

**Table 2:** Mean and standard deviation of post-operative Uncorrected and Corrected Visual Acuity expressed as logMAR, and Postoperative SE and Cylinder for the two groups.

Parameter	Group		*p*-Value ^a^
	ACRIVA	Monofocal	
UCNVA (40 cm)	0.17 ± 0.21	0.43 ± 0.11	<0.001
UCIVA (60 cm)	0.12 ± 0.18	0.39 ± 0.14	<0.001
UCDVA	0.06 ± 0.09	0.12 ± 0.13	0.888
CNVA (40 cm)	0.01 ± 0.03	0.00 ± 0.02	0.211
CDVA	−0.02 ± 0.05	−0.01 ± 0.04	0.843
SE	−0.10 ± 0.14	−0.07 ± 0.12	0.460
Cylinder	−0.19 ± 0.27	−0.15 ± 0.24	0.615

RMS = root mean square; SE = spherical equivalent; UCNVA = uncorrected near visual acuity; UCIVA = uncorrected intermediate visual acuity; UCDVA = uncorrected distance visual acuity; CNVA = corrected near visual acuity; CDVA = corrected distance visual acuity . ^a^ *p*-values corrected with false discovery rate.

**Table 3:** Mean and standard deviation of aberrometric parameters of the two groups at different time points.

Parameter (μm)	Group		*p*-Value ^a^	*p*-Value ^b^	*p*-Value ^c^
	ACRIVA	Monofocal			
RMS Total OPD			0.330	0.328	0.335
Baseline	1.80 ± 1.01	1.87 ± 0.92			
30 days	0.51 ± 0.06	0.83 ± 0.18			
90 days	0.58 ± 0.20	0.77 ± 0.17			
180 days	0.52 ± 0.21	1.02 ± 0.78			
RMS HOA			0.029 °	0.357	0.163
Baseline	0.35 ± 0.19	0.52 ± 0.25			
30 days	0.24 ± 0.06	0.37 ± 0.10			
90 days	0.28 ± 0.08	0.37 ± 0.13			
180 days	0.22 ± 0.09	0.70 ± 0.75			
RMS Coma			0.005 °	0.700	0.916
Baseline	0.12 ± 0.09	0.19 ± 0.16			
30 days	0.08 ± 0.03	0.14 ± 0.08			
90 days	0.10 ± 0.06	0.16 ± 0.07			
180 days	0.09 ± 0.07	0.18 ± 0.12			
RMS Trefoil			0.038 °	0.490	0.611
Baseline	0.27 ± 0.17	0.35 ± 0.17			
30 days	0.19 ± 0.06	0.29 ± 0.08			
90 days	0.21 ± 0.10	0.28 ± 0.12			
180 days	0.14 ± 0.08	0.52 ± 0.57			
RMS Spherical			0.046 °	0.022 °	0.962
Baseline	0.05 ± 0.09	0.09 ± 0.13			
30 days	0.03 ± 0.02	0.05 ± 0.03			
90 days	0.05 ± 0.02	0.08 ± 0.04			
180 days	0.03 ± 0.02	0.07 ± 0.08			

^a^*p*-value relative to effect of period; ^b^*p*-value relative to effect of type of IOL; ^c^*p*-value relative to interaction term (time × type of IOL). ° Significant after FDR correction.

## Data Availability

The datasets generated during and/or analyzed during the current study will be available from the corresponding author on reasonable request.
